# Representations of Older Adults’ Digital Literacy in Canadian News Media: Critical Discourse Analysis Using Unified Theory of Acceptance and Use of Technology 2

**DOI:** 10.2196/69373

**Published:** 2025-08-29

**Authors:** Alixe Ménard, Alexandra Maharaj, Sophie Harb, Sarah Fraser, Tracey O'Sullivan

**Affiliations:** 1 Interdisciplinary School of Health Sciences, Faculty of Health Sciences, University of Ottawa, 200 Lees Ave, Ottawa, ON, K1S5L5, Canada, 1 (613) 562-5800 ext. 2306; 2 LIFE Research Institute, University of Ottawa, Ottawa, ON, Canada; 3 Telfer School of Management, University of Ottawa, Ottawa, ON, Canada

**Keywords:** digital divide, media representation, older adults, disaster planning, digital technology

## Abstract

**Background:**

The transition of social and health services to digital platforms has significant implications for the inclusion and well-being of older adults. Digital literacy is a key determinant of health and equity, particularly as services shift to digital platforms. For older adults, this is crucial for accessing essential services and information, aiding in emergency resource navigation and information access.

**Objective:**

This study examines how Canadian news media portray older adults’ digital literacy, using the Unified Theory of Acceptance and Use of Technology 2 (UTAUT2) framework to guide the analysis.

**Methods:**

News media articles from Canada, published from May 4, 2023, to July 4, 2024, were analyzed. Articles were identified through database searches and manual reviews, with inclusion criteria focusing on mentions of older adults and digital literacy. Critical discourse analysis was conducted using UTAUT2 factors to interpret the media narratives.

**Results:**

Of the 103 articles reviewed, 54 met the inclusion criteria. Four key themes emerged: (1) Performance and effort expectancy: media frequently depicted older adults as needing significant help with digital technologies, indicating potential digital ageism. (2) Social influence: fearmongering narratives suggest insufficient technological skills could result in social exclusion and limited access to essential services. (3) Facilitating conditions: articles highlighted older adults’ susceptibility to digital scams and fraud, reinforcing digital ageism. (4) Hedonic motivation: media portrayals emphasized active aging, illustrating how digital tools, such as home security systems, can enhance independence and quality of life.

**Conclusions:**

Many media narratives on older adults’ digital literacy perpetuate digital ageism. The study highlights how negative portrayals of older adults’ digital skills and their exclusion from digital spaces underscore the need for more inclusive media representations. Findings suggest that media outlets could play a crucial role in shifting to more supportive portrayals of older adults’ engagement with technology. Improving media portrayals can enhance digital engagement and preparedness among aging populations, contributing to better overall quality of life.

## Introduction

### Background

Digital literacy refers to the diverse skill levels individuals possess in using digital technologies and navigating their associated risks with discernment and confidence [1]. As a determinant of health, the ability to navigate digital environments shapes access to health resources and broader health equity outcomes [2-4]. Digital literacy involves the combination of cognitive abilities and technical skills, allowing individuals to effectively use digital technology for information retrieval, critical evaluation, content creation, and communication [4]. The rapid shift of social and health services to digital platforms has profound implications for the inclusion and well-being of older adults [5].

The intersection of an aging population with rapid technological advancements has fostered the misconception that older adults are technologically incompetent [6]. This belief exemplifies digital ageism, which encompasses age-related biases in how digital technology is developed, applied, and perceived by society [7]. Digital ageism refers to the systemic exclusion or marginalization of older adults in digital environments and is embedded in both the design and dissemination of technology [8]. It has been categorized as occurring at both individual and organizational levels. At the individual level, digital ageism involves the failure to consider the specific needs, preferences, and abilities of older adults in the design and use of technology [9]. At the organizational level, it is perpetuated by a lack of training and awareness among technology developers, including website designers and service providers, leading to digital platforms that are not accessible or inclusive of older adult users [9]. These issues are further compounded by how emerging technologies, such as artificial intelligence, are developed and deployed often without adequate representation of older adults in training data or user testing processes [9]. Digital ageism fosters stereotypes that assume older adults lack digital skills [8]. In actuality, older adults possess diverse digital competencies and engage with information and communication technology in various ways [7].

Digital inclusion is essential not only for everyday activities, such as staying socially connected, but also for navigating high-stakes situations. The consequences of digital exclusion become particularly pronounced during emergencies, when timely access to digital information and services is critical. As digital ageism limits older adults’ engagement with technology, it can reduce their ability to respond effectively in times of crisis. This was especially evident during the COVID-19 pandemic, which saw a rise in the use of telehealth [10], digital banking channels [11], and web-based grocery services [12] to mitigate contact exposure [13]. More broadly, digital platforms offer invaluable resources for accessing emergency services, support networks, and relief supplies during disasters [14]. The swift dissemination of accurate and timely information, often facilitated through digital channels, is thus necessary for informed decision-making and prompt action during disasters [15].

Digital literacy equips individuals with the skills to navigate digital platforms efficiently, enabling them to access emergency alerts, evacuation routes, and safety guidelines without delay [14]. Consequently, it is essential to promote digital literacy among older adults to enhance their ability to navigate digital resources effectively, stay informed about disaster risks, and access timely assistance during a crisis [16]. Empowering older adults with digital literacy skills can foster greater resilience and preparedness within aging populations, ultimately contributing to more effective disaster response and recovery efforts [17]. Of note, digital literacy includes a broad set of competencies such as the technical skills required to use digital devices, the ability to access, evaluate and manage information, critically assess media content, and navigate digital systems such as web browsers [18]. Given this broad definition, it is reductive to assume that low digital literacy is inherent to older adults solely because they did not come of age during the digital era [18]. This stereotype promotes the falsehood that older adults are less capable of digital engagement and that technology is a privilege reserved for younger users [18]. Such assumptions diminish older adults’ self-efficacy and interest in being digitally active [19]. Therefore, it is essential to critically assess the language used when discussing older adults in digital contexts to avoid reinforcing the digital divide as a socially constructed and preventable inequity [19].

### Theoretical Framework

The Unified Theory of Acceptance and Use of Technology (UTAUT) model has previously been used to explain behavior and intentions related to the adoption of digital technology [20,21]. Technology-related behaviors are evaluated based on 5 factors that influence acceptance and use: (1) performance expectancy; (2) effort expectancy; (3) social influence; (4) facilitating conditions; and (5) age, gender, and experience [22]. In 2012, the original UTAUT model was expanded into UTAUT2, incorporating additional factors such as hedonic motivation, price value, and habit [23].

Performance expectancy refers to the perceived usefulness of digital technology [21]. Effort expectancy assesses the ease of use of the technology. Social influence evaluates the extent to which an individual’s social network values the use of digital technology. Facilitating conditions encompass the resources and support available to an individual for using the technology [21]. UTAUT suggests that behavioral intention is influenced by factors such as age, gender, and experience [21]. In UTAUT2, hedonic motivation relates to the enjoyment experienced when using digital technology [23]. Price value refers to the cognitive assessment of the balance between the perceived benefits and the monetary costs. Finally, habit describes the extent to which digital technology use is automatic (based on previous usage) [23].

In this study, we leverage UTAUT2 to evaluate news media representations of older adults’ digital literacy within the context of disaster risk management. Specifically, we investigate how newspapers depict older adults as agents with varying degrees of digital proficiency using UTAUT2 factors. Our guiding question was: What prevailing narratives surround digital literacy and older adults, and how are they depicted in news media narratives related to disaster risk management? While this focus shaped our search strategy and inclusion criteria described below, we ultimately found few disaster media articles that addressed older adults’ digital inclusion or exclusion. This absence became a significant finding in itself, prompting a broader examination of how digital literacy and older adults are framed in mainstream media more generally.

## Methods

### Positionality

As researchers, we acknowledge that our backgrounds and experiences shape our perspectives and approaches to this study. Our research team comprises 5 women, 2 of whom are aged 50 years and older, all of whom are digitally literate. These factors influence our understanding of digital literacy and our interpretation of media representations of older adults. Our collective experience as women, including those who have navigated digital landscapes for decades, informs our sensitivity to issues of age and gender bias in media portrayals. Our digital literacy enables us to critically assess the technical aspects of digital engagement, while our personal and professional experiences with media analysis and expertise in ageism research provide a nuanced lens through which we view the data. We have been conscientious in our approach to the data and remained reflexive in our interpretations by holding weekly team meetings where we discussed our relational awareness to ensure that “while the data are interpreted through the eyes and cultural standards of the researcher, the effects of the latter are monitored and mitigated” [24].

### Data Collection

We investigated digital Canadian news media articles related to older adults, digital literacy, and disaster risk management, shared from May 4, 2023, to July 4, 2024. These dates were selected to reflect the World Health Organization’s downgrade of the COVID-19 pandemic [25], allowing us to evaluate postpandemic discourse [25]. The news databases ProQuest, Factiva, and Eureka were systematically searched with the help of a Research Librarian (TR) to uncover relevant articles. The keywords related to digital literacy and disasters were informed by government documents, including the Government of Canada, the United Nations Educational, Scientific, and Cultural Organization, and the Intergovernmental Panel on Climate Change. The search strategies for each database are presented in Multimedia Appendix 1. Furthermore, the Canadian Broadcasting Corporation, the Canadian Television Network, and Radio-Canada were manually searched using the terms “seniors,” “elderly,” “older,” or “aged” in English, and “aîné,” “aînée,” “aînés,” or “aînées” in French.

Included articles met the following criteria: (1) mention of older adults; (2) mention of digital literacy; and (3) written in English or French. At the outset, we established “mention of disasters” as an inclusion criterion. However, due to the scarcity of articles meeting this criterion during the initial data collection phase, we made the decision to remove it. This adjustment is reflected as an important finding in the results section. All articles were imported into Zotero (Corporation for Digital Scholarship) and initially checked by the primary author (A Ménard). The senior author then reviewed 50% of the articles (SF), while the remaining 50% were divided among the other research team members (A Maharaj, SH, and TO), each reviewing one-third of this portion for a second check.

### Data Analysis

UTAUT2 was used to organize the data [23], and critical discourse analysis (CDA) was subsequently used for a deeper analysis of the data [26]. The UTAUT2 factors served as a priori constructs through which we organized the data deductively to represent the underlying discursive concept of digital literacy through technology acceptance in older adults [27]. In attempting to organize data according to UTAUT2 factors, we discovered that price value and habit were not discussed in media narratives, which led to their removal from our codebook. We also combined performance expectancy and effort expectancy because of their overlap, whereby articles described behavioral intention as being informed by ease of use, directly impacting perceived usefulness of technology. CDA was used to examine and interpret the construction and perpetuation of discourses [28] concerning digital literacy among older adults, particularly in the context of disaster risk management depicted in Canadian news media. CDA is based on the premise that the use of certain language is intentional, regardless of whether individuals are aware of their discursive choices [29]. Throughout our analysis, using CDA, news media language was understood as constructing social reality, not reflecting it [30]. Through this lens, we sought to understand theories of reality perpetuated in news media and relations of power encoded in the data [31] as they pertain to digital literacy among older adults. Further, CDA allowed us to examine how discourse reflects and perpetuates power structures and inequalities [29].

All authors first familiarized themselves with the data. Following this, the first author (A Ménard) conducted an inductive analysis of the data using open coding [32]. Subsequently, the coauthors (A Maharaj, SH, SF, and TO) independently coded a portion of the included articles. The research team met weekly to ensure consistency in coding, identify emerging patterns, and reach consensus on article inclusion. All codes were then grouped to produce a preliminary codebook, which was refined by the first author (A Ménard). The data were further analyzed by aligning the codes closely with the research question and organizing them deductively according to UTAUT2. Initial coding by all authors was conducted using Microsoft Excel, while the second round of coding by the first author was conducted using NVivo 12 (QSR International).

### Ethical Considerations

This study involved a scan of publicly available digital newspaper articles and did not involve human participants, personal data, or an intervention. As such, it did not require ethics approval in accordance with the Tri-Council Policy Statement (TCPS 2) and the policies of the University of Ottawa.

## Results

### Overview

We identified a total of 103 articles after the removal of duplicate studies. Despite a thorough search, no articles emerged that addressed digital literacy in the context of disaster risk management for older adults. This notable absence of relevant disaster-related narratives necessitated a shift in focus, leading to an analysis centered solely on the portrayal of digital literacy among older adults as depicted in the available news media. Following this pivot, a total of 54 articles (Multimedia Appendix 2) [33-86] met the inclusion criteria and were retained. The included articles underwent re-evaluation by 3 members of the research team (A Ménard, SF, and TO) to further confirm their eligibility for inclusion. Of the included articles, 17 were from Ontario [33-49], 16 from Quebec [50-65], 9 from British Columbia [66-74], 3 from Nova Scotia [75-77], 1 from Alberta [78], 1 from New Brunswick [79], 2 from Manitoba [80,81], and 5 were Canada-wide [82-86]. A total of 30 articles [33-40,42-45,47,48,67-78,80,81] were written in English, while the remaining 24 [41,46,50-65,79,82-86] were written in French.

Here, we present the themes emerging from media discourses, according to a combination of 5 UTAUT2 factors: (1) performance and effort expectancy (combined) (ie, how the media portrays the benefits and ease of use of digital technologies for older adults); (2) social influence (ie, the media’s depiction of the impact of social circles, such as family, friends, and societal norms, on older adults’ decisions to adopt and use digital technologies); (3) facilitating conditions (ie, how the media presents the availability of resources and support systems that enable older adults to use digital technologies), and (4) hedonic motivation (ie, how the media portrays the enjoyable aspects of digital technology use and how age-related characteristics impact older adults’ motivation to engage with digital tools).

### Theme 1: Performance and Effort Expectancy

The media discourse on digital technology and older adults revealed how narratives construct perceptions of performance and effort expectancy related to technology use within this population. The media depicted older adults as a homogeneous group with limited digital literacy, framing them with low performance expectancy and high effort expectancy. This portrayal reinforced a narrative that older adults require substantial assistance from younger generations to learn and navigate digital technologies. The media also emphasized the importance of older adults gaining digital literacy to ensure continuous or lifelong learning, shaping discourse around skill-building and access to digital technology. These narratives framed digital literacy as critical to the inclusion of older adults in society, reflecting a broader societal discourse that highlights both the anticipated benefits (performance expectancy) and perceived effort (effort expectancy) required for older adults to engage with digital technologies. Table 1 illustrates how these themes were represented in media narratives, reflecting the critical discourse on performance and effort expectancy.

**Table 1.** Performance and effort expectancy.

UTAUT2^a^ factors	Contents	Items
Performance expectancy (PE)	PE1: “[T]echnology is constantly oriented to younger and younger children, and less user-friendly for elders. Many seniors do not have computers and cell phones, but to navigate the challenges of our “modern” society, one can no longer rely on businesses or government offices having a human being at the end of a phone line, ready and available for help. (…) Seniors wait for hours on a phone call promising “customer help.” They show up at 6 AM to get lab tests (with the lab opening at 7 AM), perhaps because they are not comfortable with being, or able to be, in a “text line.” (Comox Valley Record, British Columbia) PE2: “Other types of customers are also affected [by bank closures], those who are less comfortable with technology, such as seniors, who generally prefer face-to-face contact.” (Le Droit, Ontario) PE3: “I’m a senior and do so resent all the generalities that we 65-year-olds are not very adept with the technologies. I frequently adjust my digital watch, use the cellular mobile telephone to send voice calls, drive a fuel-injected horseless carriage, program the remote on my TV, change the taped message on my answering machine, and, hard to believe, back up the family files onto 51/4” floppy disks once per month.” (Canadian Broadcasting Corporation, Ontario)	Limited performance and lifelong learning
Effort expectancy (EE)	EE1: “Cyber Seniors Technology Tutoring is a free, one-on-one tutoring program aimed at seniors who have little or no experience with technology. The program partners each participant with a community volunteer tutor. The two work together on developing basic computer, tablet or cellphone skills targeted to the learner’s specific device and goals.” (Eagle Valley News, British Columbia) EE2: “She helped me make notes, and then had me practice, so that she could see that the message got through to me.” (The Brandon Sun, Manitoba) EE3: “[T]hey have limited knowledge of or access to digital banking services.” (The Brandon Sun, Manitoba)	Personalized instruction and tailored learning goals

a UTAUT2: Unified Theory of Acceptance and Use of Technology 2.

### Theme 2: Social Influence

Media narratives surrounding digital literacy for older adults frequently used fearmongering tactics, suggesting a lack of technological skills could lead to social exclusion, difficulties in maintaining connections with grandchildren, and restricted access to essential services like telehealth. These portrayals framed digital literacy as crucial for bridging generational gaps and reinforcing intergenerational relationships, while also emphasizing its growing importance for accessing digital banking and web-based grocery services. Many articles depicted older adults as “vulnerable,” portraying them as being left behind by technological advancements. Consequently, these narratives advocated for community centers and libraries to develop programs aimed at enhancing digital literacy, helping older adults remain socially active and integrated members of society. Textbox 1 illustrates the social influence affecting older adults’ digital literacy, as depicted in these media narratives, highlighting factors such as societal expectations and community support.

Textbox 1. **Social influence (Unified Theory of Acceptance and Use of Technology 2 factor).**

Social influence (SI) captures how individuals’ behavior is shaped by the perceptions, support, and encouragement of others in their community.

- SI1. “Community centers or libraries are often places seniors would need to visit in order to get their hands on technology. However, teaching a new skill to others costs nothing, according to Garcia. ‘If you don’t teach people, they will never know how to do it, so I think it is very beneficial for the seniors community to have people guide them on ways to keep them connected.” (Richmond News, British Columbia)

- SI2. “It’s all about keeping all levels of our community connected.” (Saltwire, Nova Scotia)
- SI3. “Telehealth is becoming more accessible, many of the elderly, my grandparents included, lack computer skills to book online appointments.” (Vancouver Province, British Columbia)

Themes: community support, staying connected, and intergenerational contact.

### Theme 3: Facilitating Conditions

Media portrayals constructed older adults as needing protection from fraud or scams within the digital sphere, reflecting broader discourses of vulnerability and dependence. These portrayals underscore the necessity for digital safety measures, increased awareness of scams, and fraud prevention training specifically targeted toward this demographic. In addition, media narratives depicted older adults as “vulnerable” to loneliness and social isolation due to perceived deficiencies in digital literacy, framing social participation and connectivity as key solutions. The discourse highlighted the importance of facilitating conditions, such as access to technology, technical support, and training programs through local libraries, as crucial resources enabling older adults to engage with digital technologies. By focusing on these supportive measures, media narratives constructed a need for comprehensive systems to enhance digital literacy, reinforcing a view of older adults as needing assistance to navigate the digital landscape effectively. However, this framing of older adults as targets of digital scams may simultaneously discourage technology use and perpetuate negative stereotypes about their technological competencies compared with younger adults. Textbox 2 illustrates the facilitating conditions affecting older adults’ digital literacy as depicted in these media narratives.

Textbox 2. **Facilitating conditions (Unified Theory of Acceptance and Use of Technology 2 factor).**

Facilitating conditions (FC) refer to the resources and support available to help older adults use digital technologies. These include training programs, access to devices, and institutional support to ensure safety and reduce isolation.

- FC1: “The purpose of the workshop was to equip seniors with the knowledge and tools to protect themselves from common scams in an increasingly digital world.” (Wiarton Echo, Ontario)
- FC2: “Vancouver police are handing free mobile phones to low-income seniors to ensure they can call 911 in an emergency.” (Vancouver Sun, British Columbia)
- FC3: “Learning computer skills helps seniors overcome feelings of isolation and loneliness.” (The Chronicle Herald, Nova Scotia)
  Themes: safety, scams, and loneliness.

### Theme 4: Hedonic Motivation

In this context, hedonic motivation is defined as the enjoyment and pleasure derived from digital technology. Media depictions of older adults using digital technologies often construct a narrative of enjoyment and pleasure, reflecting the concept of active aging and its role in enhancing quality of life. The media highlighted the positive aspects of digital technology, such as its potential to support aging in place and promote independence and self-efficacy through tools like home security systems and voice-activated assistants. However, these portrayals often framed enjoyment not in terms of amusement or leisure, but through the lens of safety, control, and reassurance, reflecting a more utilitarian interpretation of hedonic motivation. These reinterpretations had age-attributed nuances, revealing how media narratives framed older adults’ interactions with digital tools based on their specific needs and challenges rather than for amusement or pleasure. For instance, difficulties in navigating digital customer service, accessing government support, and managing telehealth were common barriers depicted. This suggests a shift in how “reward” is represented for older adults in digital contexts, highlighting satisfaction derived from security and autonomy, rather than entertainment as is normally described in younger adults. These representations illustrated how media discourse on hedonic motivation intersected with practical considerations, shaping perceived motivation and engagement of older adults with digital tools. Such framings may also reflect underlying ageist assumptions that older adults engage with technology primarily for functional rather than pleasurable purposes. Textbox 3 illustrates how hedonic motivation influences older adults’ digital literacy as depicted in these media narratives.

Textbox 3. **Hedonic motivation (Unified Theory of Acceptance and Use of Technology 2 factor).**

Hedonic motivation (HM) refers to the enjoyment, satisfaction, or pleasure derived from using technology. For older adults, this motivation is often linked to enhanced quality of life, emotional comfort, and cognitive benefits.

- HM1: “Helping seniors get connected to important services and family members is essential in supporting their full participation in society and improving their quality of life.” (Vernon Morning Star, British Columbia)
- HM2: “Just having a system welcome you home by turning the lights on can be a comfort, not to mention a safety precaution.” (The Chronicle Herald, Nova Scotia)
- HM3: “By pursuing computer use, social activities, and games in both midlife (age 50‐65) and late life, benefits were noted, and a reduced risk of mild cognitive impairment was found.” (The Canadian Press, Canada-wide)
  Themes: satisfaction, safety, and cognition.

## Discussion

### Principal Findings

Our exploration of media narratives on digital literacy and older adults highlighted several key themes. Performance and effort expectancy were often portrayed negatively, with older adults seen as having limited digital skills and facing significant challenges in learning new technologies, thus necessitating extensive support. Social influence was emphasized through fearmongering, depicting older adults as “vulnerable” to social exclusion and advocating for community-based digital literacy programs to address societal expectations and support. Facilitating conditions were portrayed as crucial, with media focusing on the need for access to digital technology, technical support, and fraud prevention, though this focus on vulnerabilities may inadvertently discourage digital technology adoption by older adults. Finally, hedonic motivation was reflected in media portrayals that emphasized the enjoyment and benefits of digital technology use for enhancing independence and quality of life, while also acknowledging age-attributed challenges. Overall, media narratives both highlighted and complicated the factors influencing older adults’ digital literacy, intertwining practical and motivational aspects with underlying digital ageist undertones.

Older adults were predominantly depicted as deficient in digital literacy, reinforcing digital ageism by suggesting they will be left behind unless they adapt to new technologies [9]. These narratives often used fear tactics, emphasizing the potential loss of critical services such as banking, groceries, and health care if older adults do not embrace digital technology. In addition, there was recurring portrayal of older adults as particularly susceptible to scams and fraud, with reliance on younger adults to bridge the digital literacy gap. However, recent research has found no evidence that older adults are more likely than individuals of other ages to be victims of cyber fraud [87]. Research on susceptibility to scams indicates that older adults who are more susceptible typically have lower levels of cognition [88]. However, this finding does not apply to all older adults and may indicate ageist generalizations perpetuated by news media.

### Comparison With Previous Work

The media portrayals described above not only reinforce stereotypes about older adults’ digital competence but also highlight a critical issue: their exclusion from essential areas such as disaster risk preparedness [89]. The exclusion of older adults in the context of digital literacy and disaster risk leads to their systematic marginalization in disaster preparedness efforts [14]. In disaster scenarios, digital technology is crucial for supporting communication between individuals, their families, and health authorities; social media is often used to disseminate health information to the masses; and telehealth serves as the primary conduit for health care during public health emergencies and subsequent lockdowns [14]. As society increasingly relies on digital tools for disseminating crucial information and coordinating emergency responses, individuals with limited digital proficiency face heightened vulnerability [90]. This exclusion exacerbates their risk during disasters, underscoring the urgent need for inclusive strategies to enhance digital literacy and ensure equitable access to disaster preparedness resources [90]. As such, it is important to consider how the narrative of older adults lacking digital literacy and thus being “vulnerable” in disasters perpetuates digital ageism and deters them from meaningfully using digital technology [91].

Performance expectancy positively predicts behavioral intention, suggesting that individuals who perceive significant benefits from digital literacy are more likely to intend to use digital technology [92]. Similarly, effort expectancy is also a positive predictor, indicating that individuals who find digital technology easy to use are more likely to intend to adopt it, thereby enhancing their digital literacy [92]. That said, it is important to distinguish between ageist stereotypes and legitimate barriers that may affect older adults’ engagement with digital technology. While media portrayals often attribute digital disengagement solely to age-related decline or resistance to change, they frequently overlook key structural and design-related challenges [93]. For example, small screen sizes, complex interfaces, limited accessibility features, sensory or cognitive impairments, and the cost of digital devices or internet access can all contribute to lower digital engagement among older adults [94]. These barriers are not inherent to aging but rather reflect gaps in inclusive design, digital equity, and the need to address concerns related to privacy and safety [95]. Recognizing these factors helps reframe the issue from individual “inability” to systemic inaccessibility. Moreover, some media narratives have begun to highlight initiatives that promote inclusive digital literacy, such as community-based training programs, intergenerational tech mentoring, and age-friendly design innovations, which represent important counter-narratives to digital ageism [96,97].

However, media portrayals of older adults as lonely and socially isolated, combined with depictions of their difficulties with digital technology, can negatively impact these predictors [98]. When media frames digital technology as a crucial tool for alleviating loneliness and overcoming isolation, it highlights the perceived benefits (performance expectancy) but simultaneously underscores the challenges (effort expectancy). This dual portrayal may diminish behavioral intention by emphasizing both the urgency and complexity of adopting digital technology, potentially discouraging older adults from engaging with digital technology [99]. Social influence positively predicts behavioral intention, meaning that individuals who perceive support from important people in their social networks are more likely to intend to use digital technology [100]. Conversely, those who do not feel such support are less likely to intend to adopt digital technology [100].

Media representations of older adults as “vulnerable” to social exclusion and reliant on community centers and libraries for digital literacy workshops highlight the role of social support in digital technology adoption [101]. Facilitating conditions positively predict behavioral intention, suggesting individuals who believe in the availability of resources to aid their digital technology use are more likely to intend to use it [92]. In contrast, those who perceive a lack of such resources are less inclined to adopt digital technology [92]. Media portrayals that depict older adults as “vulnerable” to scams and frauds exacerbate concerns about internet safety and complicate their digital technology use, reinforcing the need for supportive conditions [102].

Hedonic motivation, or the enjoyment derived from using digital technology, is another positive predictor of behavioral intention [92]. Individuals who find technology enjoyable are more likely to intend to use it [92]. Our findings further align with existing literature, which suggests social perceptions of older age act as a barrier to digital technology use among this population, reinforcing stereotypes about older adults and their use of technology [91,103,104]. Notably, literature suggests that ageism influences the use and acceptance of digital technology through the inferiorization, patronage, deprioritization, and exclusion of older adults in the digital age [7,9,105,106]. Our findings contribute to the literature by highlighting how the media perpetuates digital ageism and may discourage older adults from meaningfully engaging with digital technology. Information disseminated through news media has been recognized as a potential source of negative beliefs and biased behaviors toward older adults [107]. This underscores how news media are uniquely positioned to transform narratives surrounding older adults and digital literacy. Rather than reinforcing ageist stereotypes, media outlets can foster inclusion of older adults in the digital age, promote positive representations, and support their engagement with technology, which can enhance their quality of life and participation in society.

The findings from this study also highlight how older adults are forgotten within narratives around disaster risk preparedness using digital literacy. This exclusion reflects a broader pattern of ignoring the needs of older adults in critical areas such as disaster preparedness, further perpetuating ageism and limiting their access to essential information resources. Future research should explore strategies to better integrate older adults into disaster risk management narratives and assess the impact of inclusive digital literacy initiatives on their preparedness and resilience.

### Limitations

This paper offers valuable insights into how Canadian news media portrayals of older adults and digital literacy reinforce digital ageism and marginalization of older adults in disaster risk preparedness. While our analysis highlights how certain media portrayals of older adults in digital contexts may reinforce ageist narratives, we acknowledge the risk of overinterpreting all negative depictions as inherently ageist. Some representations, such as those addressing digital scams or usability challenges, may reflect legitimate concerns rather than discriminatory assumptions. As ageism researchers, we approached the data critically but remained mindful of this interpretive tension, which we recognize as a limitation and a reflection of our analytical lens. This study benefits from a robust methodological approach, including a systematic search of multiple databases and detailed CDA using UTAUT2 [23,26]. The use of diverse media sources and languages adds depth to the findings [108]. However, limitations include the decision to constrain the focus of the study on Canadian media, which may not capture global perspectives on digital literacy for older adults in disaster risk preparedness. Future research could address these limitations by expanding the scope to include international media and by developing strategies to integrate digital literacy in older adults into disaster preparedness narratives more effectively. Furthermore, we did not include social media in our search and focused strictly on newspapers and broadcast news websites (eg, Canadian Broadcasting Corporation). Yet, we recognize that social media (eg, Instagram and Facebook) has become a key medium for news sharing [109], and has changed the production flow and delivery of news for many broadcasting corporations [110]. Future research should investigate perceptions of digital literacy in older adults as discussed on social media and explore user sentiment in the comments under posts from key news organizations.

### Conclusion

While this study was initially designed to examine media narratives at the intersection of digital literacy and disaster resilience among older adults, the limited presence of such discussions in news media led to a necessary broadening of scope. This absence, however, is a significant finding in itself, highlighting the exclusion of older adults from critical public discourse on digital preparedness during crises. This paper underscores the critical impact of media narratives on the digital literacy of older adults, revealing how these portrayals perpetuate digital ageism and contribute to the marginalization of older adults in essential areas such as disaster risk preparedness. By highlighting the negative portrayal of older adults’ digital competencies and their exclusion from disaster preparedness discussions, the study demonstrates the need for a shift in media representation. The findings suggest media outlets can fulfill a pivotal role in transforming the narrative, promoting more inclusive and supportive portrayals of older adults’ engagement with digital technology. Future research should seek to broaden the scope to include international perspectives and explore strategies for more effective integration of older adults into digital literacy and disaster preparedness narratives.

## Supplementary material

10.2196/69373 Multimedia Appendix 1 Search strategy for older adults and digital literacy.

10.2196/69373 Multimedia Appendix 2 References for newspaper articles.

## Abbreviations

CDA: critical discourse analysis
UTAUT: Unified Theory of Acceptance and Use of Technology
UTAUT2: Unified Theory of Acceptance and Use of Technology 2
