# Supplementary material for: Representations of Older Adults’ Digital Literacy in Canadian News Media: Critical Discourse Analysis Using Unified Theory of Acceptance and Use of Technology 2
Source: JMIR Aging. 2025 Aug 29;8:e69373. doi: 10.2196/69373 (PMC12396828; doi:10.2196/69373)
Supplement: Multimedia Appendix 1 [file aging-v8-e69373-s001.docx]

Multimedia Appendix 1

**Appendix 1.** Search strategy for older adults and digital literacy.

| **Older adults** |
| --- |
| **ProQuest** |
| (elderly OR ((older OR aged OR aging OR ageing OR elder* OR sixties OR seventies OR eighties OR nineties) n/3 (adult* OR person* OR population OR people OR m?n OR wom?n OR demographic*)) OR seniors OR elders OR retiree* OR retired OR geriatric* OR “advanced age” OR sexagenarian* OR septuagenarian* OR octogenarian* OR nonagenarian* OR centenarian* OR supercentenarian* OR ((6? OR 7? OR 8? OR 9? OR 10? OR sixty* OR seventy* OR eighty* OR ninety* OR hundred) p/3 (old))) |
| **Factiva** |
| English keywords:  (elderly OR ((older OR aged OR aging OR ageing OR elder* OR sixties OR seventies OR eighties OR nineties) near4 (adult* OR person* OR population OR people OR man OR woman OR men OR women OR demographic*)) OR seniors OR elders OR retiree* OR retired OR geriatric* OR “advanced age” OR sexagenarian* OR septuagenarian* OR octogenarian* OR nonagenarian* OR centenarian* OR supercentenarian* OR ((65 OR 66 OR 67 OR 68 OR 69 OR 70 OR 71 OR 72 OR 73 OR 74 OR 75 OR 76 OR 77 OR 78 OR 79 OR 80 OR 81 OR 82 OR 83 OR 84 OR 85 OR 86 OR 87 OR 88 OR 89 OR 90 OR 91 OR 92 OR 93 OR 94 OR 95 OR 96 OR 97 OR 98 OR 99 OR 100 OR 101 OR 102 OR 103 OR 104 OR 105 OR 106 OR 107 OR 108 OR 109 OR 110 OR 111 OR 112 OR 113 OR 114 OR 115 OR sixty* OR seventy* OR eighty* OR ninety* OR hundred) adj4 (old)))  French keywords: (aine* OR vieillesse OR ((age* OR ain* OR retraite* OR vieillissant*) near4 (adulte* OR personne* OR population OR homme* OR femme*)) OR retraite* OR geriatr* OR “age avance” OR soixantaine OR sexagenaire***** OR septuagenaire* OR octogenaire* OR nonagenaire* OR centenaire* OR supercentenaire* OR ((65 OR 66 OR 67 OR 68 OR 69 OR 70 OR 71 OR 72 OR 73 OR 74 OR 75 OR 76 OR 77 OR 78 OR 79 OR 80 OR 81 OR 82 OR 83 OR 84 OR 85 OR 86 OR 87 OR 88 OR 89 OR 90 OR 91 OR 92 OR 93 OR 94 OR 95 OR 96 OR 97 OR 98 OR 99 OR 100 OR 101 OR 102 OR 103 OR 104 OR 105 OR 106 OR 107 OR 108 OR 109 OR 110 OR 111 OR 112 OR 113 OR 114 OR 115 OR soixant* OR quatre-vingt* OR cent) adj4 (ans))) |
| **Eureka** |
| English keywords: (elderly \| ((older \| aged \| aging \| ageing \| elder* \| sixties \| seventies \| eighties \| nineties) %4 (adult* \| person* \| population \| people \| man \| woman \| men \| women \| demographic*)) \| seniors \| elders \| retiree* \| retired \| geriatric* \| “advanced age” \| sexagenarian* \| septuagenarian* \| octogenarian* \| nonagenarian* \| centenarian* \| supercentenarian* \| ((65 \| 66 \| 67 \| 68 \| 69 \| 70 \| 71 \| 72 \| 73 \| 74 \| 75 \| 76 \| 77 \| 78 \| 79 \| 80 \| 81 \| 82 \|83 \| 84 \| 85 \| 86 \| 87 \| 88 \| 89 \| 90 \| 91 \| 92 \| 93 \| 94 \| 95 \| 96 \| 97 \| 98 \| 99 \| 100 \| 101 \| 102 \| 103 \| 104 \| 105 \| 106 \| 107 \| 108 \| 109 \| 110 \| 111 \| 112 \| 113 \| 114 \| 115 \| sixty* \| seventy* \| eighty* \| ninety* \| hundred) $4 (old)))  French keywords: (aine* \| vieillesse \| ((age \|ages \| agee* \| aine* \| retraite* \| vieillissant*) %4 (adulte* \| personne* \| population \| homme* \| femme*)) \| retraite* \| geriatr* \| “age avance” \| soixantaine \| sexagenaire* \| septuagenaire* \| octogenaire* \| nonagenaire* \| centenaire* \| supercentenaire* \| ((65 \|66 \| 67 \| 68 \| 69 \| 70 \| 71 \| 72 \| 73 \| 74 \| 75 \| 76 \| 77 \| 78 \| 79 \| 80 \| 81 \| 82 \| 83 \| 84 \| 85 \| 86 \| 87 \| 88 \| 89 \| 90 \| 91 \| 92 \| 93 \| 94 \| 95 \| 96 \| 97 \| 98 \| 99 \| 100 \| 101 \| 102 \| 103 \| 104 \| 105 \| 106 \| 107 \| 108 \| 109 \|110 \| 111 \| 112 \| 113 \| 114 \| 115 \| soixant* \| quatre-vingt* \| cent) $4 (ans))) |
| **Digital literacy** |
| **ProQuest** |
| ((digital OR technolog* OR computer* OR media OR ICT OR online OR cyber OR internet OR web-based) n/4 (literacy OR literate OR skill* OR knowledg* OR capabilit* OR abilit* OR proficien* OR able)) |
| **Factiva** |
| English keywords: ((digital OR technolog* OR computer* OR media OR ICT OR online OR cyber OR internet OR web-based) near4 (literacy OR literate OR skill* OR knowledg* OR capabilit* OR abilit* OR proficien* OR able))  French keywords: ((numerique* OR technolog* OR ordinateur* OR media* OR TIC OR “en ligne” OR cyber OR internet OR “le Web” OR informatique*) near4 (litteratie OR competen* OR connaissance* OR maitris* OR capable* OR capac* OR aptitude*)) |
| **Eureka** |
| English keywords: ((digital \| technolog* \| computer* \| media \| ICT \| online \| cyber \| internet \| web-based) %4 (literacy \| literate \| skill* \| knowledg* \| capabilit* \| abilit* \| proficien* \| able))  French keywords: ((numerique* \| technolog* \| ordinateur* \| media* \| TIC \| “en ligne” \| cyber \| internet \| “le Web” \| informatique*) %4 (litteratie \| competen* \| connaissance* \| maitris* \| capable* \| capac* \| aptitude*)) |
| **Disaster risk** |
| **ProQuest** |
| (risk* OR crisis OR crises OR emergenc* OR hazard* OR disaster* OR outbreak* OR catastroph* OR insecur* OR damag* OR sensitivity OR exposure OR stress*) |
| **Factiva** |
| English keywords: (risk* OR crisis OR crises OR emergenc* OR hazard* OR disaster* OR outbreak* OR catastroph* OR insecur* OR damag* OR sensitivity OR exposure OR stress))  French keywords: (risque* OR crise* OR urgence* OR danger* OR sinistre* OR epidem* OR catastroph* OR securit* OR insecurit* OR endommage* OR dommage* OR sensibilite OR exposition OR perturbation*)) |
| **Eureka** |
| English keywords: (risk* \| crisis \| crises \| emergenc* \| hazard* \| disaster* \| outbreak* \| catastroph* \| insecur* \| damag* \| sensitivity \| exposure \| stress))  French keywords: (risque* \| crise* \| urgence* \| danger* \| sinistre* \| epidem* \| catastroph* \| securit* \| insecurit* \| endommage* \| dommage* \| sensibilite \| exposition \| perturbation*)) |
