# Supplementary material for: Representations of Older Adults’ Digital Literacy in Canadian News Media: Critical Discourse Analysis Using Unified Theory of Acceptance and Use of Technology 2
Source: JMIR Aging. 2025 Aug 29;8:e69373. doi: 10.2196/69373 (PMC12396828; doi:10.2196/69373)
Supplement: Multimedia Appendix 2 [file aging-v8-e69373-s002.docx]

Multimedia Appendix 2. References for newspaper articles mentioned in the study.

Ontario

33. Carol Baldwin LJIR. Why Governments Should Support Seniors Clubs. The Canadian Press. Published online July 28, 2023. https://nouveau-eureka-cc.proxy.bib.uottawa.ca/Link/KO00451T_1/news·20230728·CP·3585f2ed8bf2408f96d3d31eaec9a9f8

34. Leon C. $255K in federal funds support London projects for seniors. Strathroy Age Dispatch. July 6, 2023:A8.

35. Become a lifeguard at any age with new Clarington program. Clarington This Week. July 4, 2023:1.

36. Kerr P. Bruce County Public Library holds anti-scam workshop. Wiarton Echo. December 12, 2023:A9.

37. City launches Healthy Aging Passport program. Brantford Expositor. June 13, 2023:A3.

38. Edwards J. Collingwood’s Breaking Down Barriers sees massive growth thanks to pandemic pivoting: “I think it propelled us”; More than 3,000 people accessed services in 2022. Collingwood Connection. September 22, 2023:1.

39. Lubna El Elaimy LJIR. Cybercriminals target Burnaby residents. The Canadian Press. Published online March 7, 2024. https://nouveau-eureka-cc.proxy.bib.uottawa.ca/Link/KO00451T_1/news·20240307·CP·f95d232cac154cc082cdd0232b063447

40. Federal grant for Cambridge older adult home will target resident programs. Cambridge Times. July 13, 2023:1.

41. Fermetures de services et de guichets bancaires : des clientèles vulnérables. Le Droit Online. http://global.factiva.com/redir/default.aspx?P=sa&an=CSULTW0020240223ek2n000gw&cat=a&ep=ASE. February 23, 2024. Accessed April 2, 2024.

42. Guelph programs for seniors get $165K from feds. Guelph Mercury-Tribune. June 2, 2023:0.

43. Brierley A. Helping older adults feel less isolated and lonely this holiday season; National Institute on Ageing report finds as many as 41% of Canadians aged 50 years and older are at risk of social isolation. Barrie Advance. December 12, 2023:1.

44. Morga A. How to talk to older people in your life about scams. The Canadian Press. Published online November 10, 2023. https://nouveau-eureka-cc.proxy.bib.uottawa.ca/Link/KO00451T_1/news%c2%b720231110%c2%b7CP%c2%b7b1db3fcc863f1352b9f559b86c7be376

45. Port Colborne program helps seniors age well. Port Colborne Leader. June 28, 2023:1.

46. Dubuc C. Un projet pour aider les retraités à réintégrer le marché du travail dans l’est ontarien. ICI Radio-Canada - Ottawa-Gatineau (site web). Published online July 3, 2023. https://nouveau-eureka-cc.proxy.bib.uottawa.ca/Link/KO00451T_1/news·20230703·CSW·006

47. CBC. Seniors, caregivers and entrepreneurs turn to tech as Canada&apos;s population gets older than ever. CBC News. https://www.cbc.ca/news/canada/toronto/agetech-innovation-week-brings-seniors-caregivers-entrepreneurs-together-1.7007985. October 27, 2023. Accessed August 7, 2025.

48. Seniors Community Grants total $90,000 for programs in Lanark-Frontenac-Kingston. Perth Courier. June 2, 2023:1.

49. Canadore College is leading the way to healthier aging. North Bay Nugget. March 2, 2024:A2.

Quebec

50. 100 000$ pour les ainés de Beauport-Limoilou. Métro Québec (La Cité / Limoilou / Vanier, QC) (site web) - Metro Québec. Published online May 26, 2023. https://nouveau-eureka-cc.proxy.bib.uottawa.ca/Link/KO00451T_1/news·20230526·CHQ·1119988_7630645279_12117478

51. Radio-Canada. Des cours pour améliorer la santé cognitive des aînés à Baie-Comeau. ICI Radio-Canada - Côte-Nord (site web). Published online September 12, 2023. https://nouveau-eureka-cc.proxy.bib.uottawa.ca/Link/KO00451T_1/news·20230912·CRB·006

52. spéciale DBC. Gillou, 78 ans, tiktokeur. La Presse. http://global.factiva.com/redir/default.aspx?P=sa&an=CSUCY00020240121ek1l000rt&cat=a&ep=ASE. January 21, 2024. Accessed March 19, 2024.

53. L’AQDR Jonquière veille aux intérêts des aînés. Le Quotidien (Saguenay, QC) (tablette). Published online December 16, 2023. https://nouveau-eureka-cc.proxy.bib.uottawa.ca/Link/KO00451T_1/news·20231216·TQT·dglycupspvdtlosuvckqo7tiwq

54. Estrie L d’AQDR. La fracture numérique : un enjeu crucial pour les personnes aînées. Journal de rue de l’Estrie. December 1, 2023:8.

55. Le guichet automatique, ce nouveau «Serpuarien». Le Nouvelliste (Trois-Rivières, QC) (tablette). Published online February 5, 2024. https://nouveau.eureka.cc/Link/KO00451T_1/news·20240205·TNV·oq5h7vr26rfhjiakjxrtm6a2my

56. DESCÔTEAUX LJ de MD. LES AÎNÉS SERONT PLUS DUREMENT AFFECTÉS PAR CE CHANGEMENT. Journal de Québec. November 4, 2023:4.

57. Vitrine L. LGBTQ passé 50 ans : de l’angoisse aux solutions. Le Quotidien (Saguenay, QC) (tablette). Published online May 13, 2023. https://nouveau-eureka-cc.proxy.bib.uottawa.ca/Link/KO00451T_1/news·20230513·TQT·d2smb6tefjbe3fqmbccqrzlbq4

58. Littéracie numérique : nouveau projet pour prévenir les inégalités. L’Écho La Tuque - Haut St-Maurice Online. http://global.factiva.com/redir/default.aspx?P=sa&an=CSUWQA0020240218ek2i0008d&cat=a&ep=ASE. February 18, 2024. Accessed March 19, 2024.

59. Longueuil : penser la cité pour les aînés. Le Courrier du Sud. http://global.factiva.com/redir/default.aspx?P=sa&an=CSUWQCO020240118ek1i0005m&cat=a&ep=ASE. January 18, 2024. Accessed March 21, 2024.

60. Éric Faucher LN. Nouvelle vague de fraudes ciblant les aînés à Trois-Rivières. Le Nouvelliste (Trois-Rivières, QC) (tablette). Published online December 6, 2023. https://nouveau.eureka.cc/Link/KO00451T_1/news·20231206·TNV·44pfzz4isvb43le54ykrafi2ye

61. BÉRUBÉ G. Responsable d’être escroqué. Le Devoir. Published online February 17, 2024:A9.

62. ARCHAMBAULT LJ de MH. SANTÉ : DES AÎNÉS DE PLUS EN PLUS DÉMENTS. Journal de Montréal. October 20, 2023:7.

63. Brunet L. Techno-maltraitance Notre vie quotidienne s’est déplacée vers le monde des transactions « en ligne ». Le Devoir. Published online June 30, 2023:B9.

64. Une entreprise granbyenne veut combattre l’exclusion numérique. Granby Express Online. http://global.factiva.com/redir/default.aspx?P=sa&an=CSUCNQ0020231227ejcr00004&cat=a&ep=ASE. December 27, 2023. Accessed March 21, 2024.

65. Presse L. Les aînés qui fréquentent internet réduiraient leur risque de démence. Le Quotidien (Saguenay, QC) (tablette). Published online May 13, 2023. https://nouveau-eureka-cc.proxy.bib.uottawa.ca/Link/KO00451T_1/news·20230513·TQT·6isqoatwtvat3awcb6prjii6bi

British Columbia

66. Cyber Seniors tutoring in Sicamous. Eagle Valley News. September 21, 2023:2.

67. Leung V. Digital connection: Keeping seniors up-to-date with online news. Richmond News. http://global.factiva.com/redir/default.aspx?P=sa&an=RCMNW00020231219ejci00008&cat=a&ep=ASE. December 18, 2023. Accessed March 21, 2024.

68. Pass J. Honour and help our elders – benefit is it just might change your life. Eagle Valley News. October 19, 2023:4.

69. Smith J. Is it your year to volunteer? Vernon Morning Star. January 18, 2024:18.

70. Mccrae D. My family has been left by the health care wayside. Vancouver Province. December 6, 2023:A9.

71. Seyd J. Ottawa grants $732K to help seniors in West Vancouver. North Shore News. http://global.factiva.com/redir/default.aspx?P=sa&an=NSRNW00020240310ek3900004&cat=a&ep=ASE. March 9, 2024. Accessed March 19, 2024.

72. Scammers target seniors. Here’s how to protect them. Burns Lake Lakes District News. January 31, 2024:4.

73. McManus T. Today is World Elder Abuse Awareness Day in New Westminster. New Westminster Record. http://global.factiva.com/redir/default.aspx?P=sa&an=NWSTR00020230616ej6g00004&cat=a&ep=ASE. June 16, 2023. Accessed March 19, 2024.

74. VPD hands out free 911-only cellphones; In brief. Vancouver Sun. September 21, 2023:A6.

Nova Scotia

75. Campbell F. “We paid our dues”: Senior calls for government help to keep aging residents at home. Saltwire - Cape Breton. http://global.factiva.com/redir/default.aspx?P=sa&an=CSUWWQ0020240130ejc8001gt&cat=a&ep=ASE. December 8, 2023. Accessed April 2, 2024.

76. Connors C. Ding-dongs and blue cheese sauce: Tech Tea with Teens helps Cape Breton seniors navigate digital world. Saltwire - Nova Scotia. http://global.factiva.com/redir/default.aspx?P=sa&an=CSUHHW0020240305ek340008d&cat=a&ep=ASE. March 4, 2024. Accessed March 20, 2024.

77. LEBLANC KFL. Technology helps many seniors overcome isolation. The Chronicle Herald (Halifax, NS). Published online September 26, 2023:Provincial_A5.

Alberta

78. Sethi C. Comment: Coming together, literally, can solve our housing crisis. Western Investor. http://global.factiva.com/redir/default.aspx?P=sa&an=WSTIVN0020230624ej6o00001&cat=a&ep=ASE. June 24, 2023. Accessed March 25, 2024.

New Brunswick

79. Restigouche: un site web pour le rayonnement des aînés. Acadie Nouvelle. Published online July 1, 2023:8.

Manitoba

80. MCDOUGALL M. Students help seniors with tech skills. The Brandon Sun (MB). Published online June 10, 2023:A2.

81. IANTORNO M. Who winds up left behind when retail is automated? The Brandon Sun. February 16, 2024:A6.

Canada-wide

82. AGE-WELL, en collaboration avec le Réseau canadien des soins aux personnes fragilisées, annonce 17 projets de recherche dans le cadre du Programme de financement Catalyst pour le vieillissement en santé. Canada NewsWire (français). Published online June 8, 2023. https://nouveau-eureka-cc.proxy.bib.uottawa.ca/Link/KO00451T_1/news·20230608·FW·0c6258

83. Le gouvernement du Canada finance 112 projets au Manitoba pour assurer l’autonomie des aînés au sein de leur communauté. Canada NewsWire (français). Published online May 23, 2023. https://nouveau-eureka-cc.proxy.bib.uottawa.ca/Link/KO00451T_1/news·20230523·FW·0c4186

84. Les banques répondent aux besoins financiers uniques des aînés. Canada NewsWire (français). Published online June 5, 2023. https://nouveau-eureka-cc.proxy.bib.uottawa.ca/Link/KO00451T_1/news·20230605·FW·0c4862

85. MONTRÉAL-NORD SE MOBILISE POUR AMÉLIORER LA QUALITÉ DE VIE DE SES AÎNÉ.E.S. Canada NewsWire (français). Published online June 8, 2023. https://nouveau-eureka-cc.proxy.bib.uottawa.ca/Link/KO00451T_1/news·20230608·FW·0c3914

86. Ève Ménard I de journalisme local. Vieillir, ailleurs dans le monde. La Presse Canadienne - Le fil radio. Published online September 28, 2023. https://nouveau-eureka-cc.proxy.bib.uottawa.ca/Link/KO00451T_1/news·20230928·HR·9be629de6a3b465e8207dcdb7aa38d75
